# Transition of Living Arrangement and Cognitive Impairment Status among Chinese Older Adults: Are They Associated?

**DOI:** 10.3390/medicina57090961

**Published:** 2021-09-12

**Authors:** Yen-Han Lee, Chia-Hung Lin, Jia-Ren Chang, Ching-Ti Liu, Mack Shelley, Yen-Chang Chang

**Affiliations:** 1 Department of Public Health and Sports Medicine, McQueary College of Health and Human Services, Missouri State University, Springfield, MO 65897, USA; 2 Department of Nursing, Taipei Veterans General Hospital, Taipei City 112, Taiwan; hong76322@gmail.com (C.-H.L.); joy50422@gmail.com (J.-R.C.); 3 Department of Biostatistics, School of Public Health, Boston University, Boston, MA 02118, USA; ctliu@bu.edu; 4 Department of Political Science, Iowa State University, Ames, IA 50011, USA; mshelley@iastate.edu; 5 Department of Statistics, Iowa State University, Ames, IA 50011, USA; 6 Center for General Education, National Tsing Hua University, Hsinchu City 300, Taiwan

**Keywords:** living arrangement, cognitive function, older adults, China, multistate survival analysis

## Abstract

*Background and Objectives:* Living arrangement is a crucial factor for older adults’ health. It is even more critical for Chinese older adults due to the tradition of filial piety. With the aging of China’s population, the prevalence of cognitive impairment among older adults has increased. This study examines the association between living arrangement transition and cognitive function among Chinese older adults. *Materials and Methods:* Using three waves of the Chinese Longitudinal Healthy Longevity Survey (CLHLS; 2008–2009, 2011–2012, and 2014), we analyzed data for older adults (age ≥ 65) who lived with other household members and reported good cognitive function or mild cognitive impairment when they participated in the survey. Multistate Cox regression was employed to study changes in cognitive function. *Results:* Older adults who transitioned to living alone had lower risk of cognitive impairment (hazard ratio (HR) = 0.66, 95% CI: 0.52, 0.83; *p* < 0.01), compared with those who continued to live with other household members. Moving into an institution was also not associated with cognitive impairment. *Conclusions:* With older adults’ transition to living alone, public health practitioners or social workers might educate them on the benefits of such a living arrangement for cognitive function.

## 1. Introduction

### 1.1. Background

The number of people on the planet aged 60 years and older is expected to reach 2 billion by 2050 [1]. In the process of aging, cognitive impairment becomes one of the most common health problems for older adults [2,3,4,5]. Cognitive impairment is defined as critical cognitive changes associated with aging, which lead to deteriorating performance of memory, attention, and higher-level cognitive functions such as controlling, reasoning, evaluation, and organization [3]. Decline in cognitive function among older adults also translates into higher public expenditures. For example, the cost of caring for older adults with cognitive impairment is estimated to reach USD 2 trillion annually by 2030 [5]. This number is expected to grow in the next few decades.

The growing population of older adults around the world aggravates the financial burden on healthcare systems, with China being no exception. Cognitive impairment is an important health issue among Chinese older adults [6], which not only deteriorates the quality of life and the overall health condition of older adults but also leads to higher healthcare costs. For example, the estimated total annual cost of treating dementia and cognitive impairment in China increased from USD 0.9 to 47.2 billion between 1990 and 2010 [7]. Therefore, it is becoming imperative for public health practitioners to lower the prevalence of cognitive impairment among Chinese older adults.

Changing living arrangements may prove an effective solution to lower the incidence of cognitive impairment and other health issues among older adults. Cognitive impairment and living arrangement transition are associated with increased risk of mortality in later life [8,9]. To delay or slow the progression of cognitive impairment, it is important to unpack the complex relationship between living arrangement transition and cognitive health [10,11,12,13,14]. Older adults living with a spouse, children, unrelated persons, or alone after having a family represent various types of living arrangement transition [15]. In other words, living arrangement is important for the survival and well-being of older adults [16,17,18], since it provides a critical social support network [19,20].

### 1.2. Literature Gaps

The existing research on living arrangements and health conditions contains a number of gaps. Not all study results support the claim that living arrangement helps prevent adverse health conditions among older adults. For instance, some studies have shown that older adults who live with adult children or within multigenerational households are more likely to be disabled [21,22]. Furthermore, older adults living in multigenerational households had significantly poorer cognitive function [13], decreased independence, and faster age-related loss of physical ability. At the same time, conflicts between older adults and other household members were shown to lead to increased risk of poor health and mortality [23,24,25]. Furthermore, older adults who lived in an institution or moved into one after living with family faced a greater risk of dying, compared with older adults continuing to live with family [9]. In addition, older adults living with family had a lower mortality rate than those living alone because the former received physical and psychological support in their daily care [26]. Older adults who lived with family also had higher odds of reporting better sleep quality than those who lived alone [27]. The literature gaps regarding living arrangements among older adults demonstrate the need for further research.

A prior study demonstrated that people living alone in later life were not at greater risk of cognitive impairment because they were not prevented from seeing friends and did not experience greater social loneliness, but instead were more likely to engage in regular social activity [10]. However, the literature also presents opposite accounts. Similar to those living with family members, older adults living alone may also develop health issues due to the living arrangement. For instance, living alone was associated with increased risk of cognitive impairment [28]. Against the backdrop of an ongoing social transition in China, it is necessary to address this research gap by identifying optimal living arrangements and thus help reduce cognitive impairment among Chinese older adults.

### 1.3. Purpose of the Study

Research on the long-term relationship between living arrangement transition and cognitive impairment remains limited, reflecting in part the lack of reliable longitudinal data. In addition, endogeneity may pose an inevitable challenge with cross-sectional study designs [13]. Moreover, previous research did not evaluate changes in living arrangement during the follow-up period [11]. Finally, there have been few studies connecting changes of cognitive function with living arrangement transitions. Previous research studies have focused primarily on Singapore [13], Europe [10,12], and Japan [11], where living arrangements may differ from those in China and other emerging economies.

As social and household structures have been evolving rapidly around the globe, the living arrangements of older adults have been a dynamic rather than static phenomenon [29]. However, knowledge regarding the association between living arrangement transition and cognitive changes among older adults is unknown. This is especially so in the case of China, where living with family members is very common for older adults. Addressing this research gap, the present study uses the Chinese Longitudinal Healthy Longevity Survey (CLHLS) and a multistate survival analytic approach to examine the association between living arrangement transition and changes in cognitive impairment among Chinese older adults. We hypothesize that living arrangement transition is negatively associated with cognitive impairment among Chinese older adults.

## 2. Methods and Materials

### 2.1. Study Sample

We extracted data from the 2009 (collected between 2008 and 2009), 2012 (collected between 2011 and 2012), and 2014 waves of the CLHLS. The CLHLS dataset, established by international investigators at the Center for the Study of Aging and Human Development at Duke University, is a nationally representative survey on healthy human longevity and oldest-old mortality. The CLHLS questionnaire covers a wide array of measurements including health, disability, demographic, family, socioeconomic, and behavioral risk variables. CLHLS researchers conducted face-to-face interviews with randomly selected participants, with the surveyed regions covering about 85% of the population from 631 counties and cities in 22 provinces and mega cities of China [30] (later survey waves include 23 Chinese provinces and mega cities). Informed consent was obtained from study participants prior to interviews. The CLHLS data quality is generally good, with high reliability and validity of measurements [31]. Because this study used a secondary and de-identified dataset from the public domain, the Institutional Review Board approvals were not required at the authors’ institutions. Zeng provides further information regarding this dataset [30].

**Figure 1:**
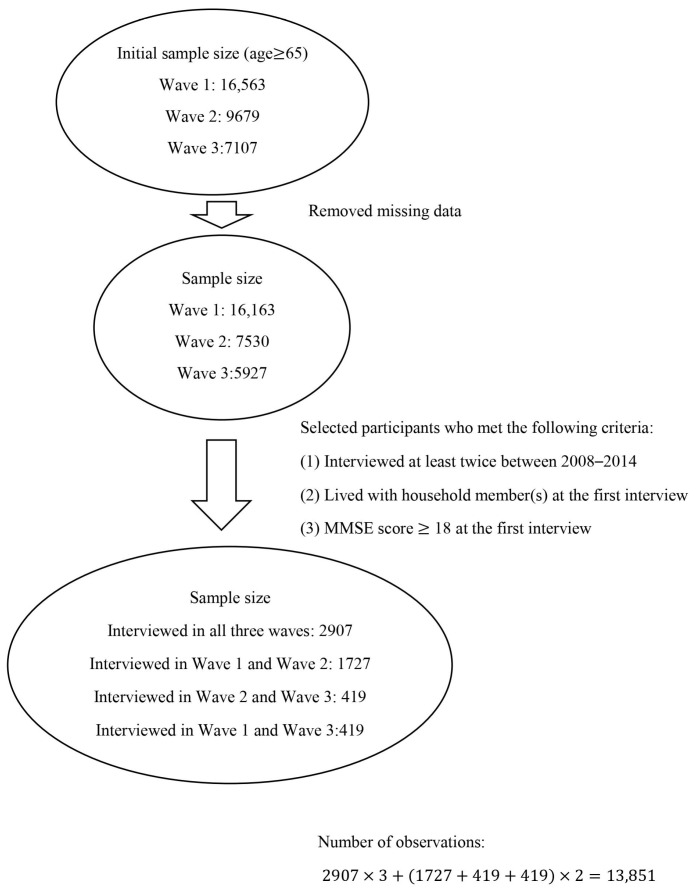
Diagram of study sample selection procedure (*n* = 13,851).

We selected older adults who were at least 65 years old when they joined the survey (age ≥ 65). Our participant selection was based on three major criteria: (1) older adults who were interviewed at least twice between the 2008–2009 wave and the 2014 wave; (2) older adults who lived with household member(s) at the time of the first interview; and (3) older adults who had no or mild cognitive impairment at the time of the first interview’s mini-mental state examination (MMSE score ≥ 18). In addition, we retained only participants who fully answered all relevant questions. With the above selection criteria, the final study sample included 13,851 observations (*n* = 13,851). Figure 1 shows the process for selecting the final study sample.

### 2.2. Primary Predictor

The primary predictor of this research, a categorical variable, was living arrangement transition among older adults. Its categories included older adults who continued to stay with other household members, became alone, and moved to an institution.

### 2.3. Outcome

In this analysis, we used MMSE scores from the CLHLS questionnaire to measure the cognitive function of older adults. Two categories of MMSE scores were employed for data analysis: good cognitive function or mild cognitive impairment (MMSE score greater or equal to 18) and moderate or severe cognitive impairment (MMSE score less than or equal to 17). We coded MMSE score 0 = good cognitive function or mild cognitive impairment, and 1 = moderate or severe cognitive impairment. All MMSE scores were measured by the CLHLS investigators. We used MMSE score = 18 as a cutoff point because a previous study showed that a cutoff between 18 and 21 might provide the highest accuracy of MMSE in detecting cognitive impairment [32], and a recent study adopted a MMSE score between 18 and 24 as mild dementia [33].

### 2.4. Covariates

We also selected a set of sociodemographic covariates for statistical analysis. Participants’ age (65–80, 81–95, and above 95; measured in years) and sex (male, female) were used to describe the biological characteristics of older adults. Older adults’ marital status (married, others [including older adults who were divorced, widowed, or not married]), formal education (no, yes), and residential area (urban, rural), were included in the analysis.

Furthermore, we picked several health-related measurements describing older adults’ health condition and well-being: smoking status (no, yes), alcohol use status (no, yes), number of times suffering from chronic conditions that required inpatient treatments in the past two years (none, 1–2 times, and above 2 times), self-rated life satisfaction (good, neutral, bad, and not able to answer), self-rated health status (good, neutral, bad, and not able to answer), and self-rated sleep quality (good, bad).

### 2.5. Statistical Analysis

To study changes in cognitive function among Chinese older adults, we conducted multistate survival analysis employing Cox regression. The multistate analytical approach allowed us to examine differences in cognitive function over time [34,35]. These changes included two stages, as we reported in the main results: (a) from good cognitive function or mild cognitive impairment to moderate or severe cognitive impairment, and (b) from moderate or severe cognitive impairment to moderate or severe cognitive impairment. Hazard ratios (HRs) and 95% confidence intervals (95% CIs) were reported in Section 3. Regression tests were two-tailed with a level of significance of 0.05 (*p-value* < 0.05), controlling for the aforementioned covariates. Statistical analysis was conducted using R software (version 3.6.2) with its package “survival” for multistate survival analysis [36].

## 3. Results

### 3.1. Descriptive Statistics

Table 1 shows MMSE scores for the study sample (*n* = 13,851), including overall sample characteristics and group characteristics. Almost 93% of older adults stayed with other household members, 6.6% became alone, and fewer than 1% moved to an institution. Most of these adults were below 95 years old, male, and married. Approximately 51% of them received formal education and 53% resided in rural areas. Most did not smoke, did not use alcohol, and did not suffer from chronic conditions that required inpatient treatments in the past two years. Approximately 63% and 64% of the older adults reported good life satisfaction and sleep quality, respectively. Furthermore, 48.8% reported good health.

### 3.2. Association between Living Arrangement Transition and Changes of Cognitive Function among Chinese Older Adults

Table 2 shows the results of the multistate Cox regression model regarding the association between living arrangement transition and changes of cognitive function among Chinese older adults. The individuals who became alone had a lower chance of deteriorating cognitive function (from good cognitive function or mild cognitive impairment to moderate or severe cognitive impairment; HR = 0.66, 95% CI: 0.52, 0.83; *p* < 0.01), compared with those who continued to live with other household members. However, this analysis found no significant changes of cognitive function among older adults who moved into an institution (*p* > 0.05).

Some results from covariates should also be noted. Older age groups were associated with deteriorating cognitive function (all *p* < 0.01) and worse cognitive impairment (all *p* < 0.05). Participants’ sex was not associated with deteriorating cognitive function. Non-married individuals had higher rates of deteriorating cognitive function (HR = 1.72, 95% CI: 1.45, 2.05; *p* < 0.01), compared with married respondents. In addition, better-educated older adults had a 45% lower chance of deteriorating cognitive function (HR = 0.55, 95% CI: 0.46, 0.65; *p* < 0.01), compared with less-educated participants. Older adults who reported poor life satisfaction and self-rated health status also had higher likelihood of experiencing deteriorating cognitive function (all *p* < 0.01), unlike participants who reported good life satisfaction.

## 4. Discussion

This research examined the association between living arrangement transition and changes in cognitive function among Chinese older adults. Toward this end, we conducted secondary analysis of a large longitudinal study sample and estimated multistate survival model. We found that older adults with good cognitive function or mild cognitive impairment who transitioned to living alone were less likely to suffer from deteriorating cognitive impairment, compared with those continuing to live with other household members. Older adults who moved to an institution also had lower likelihood of cognitive decline based on the calculated ratio, but the observed association did not reach a statistical significance. 

Our findings are consistent with previous studies suggesting that older adults who live alone generally do not experience declining cognitive function [10,11,37,38,39]. Although older adults living alone were isolated from family, they were not separated from friends. Therefore, such older adults did not experience more social loneliness and were more likely to engage in regular social activity [10]. The association between living alone and cognitive function may reflect the protective role of social engagement rather than that of social support [37].

This study focused on the transition of older adults’ living arrangements. The health advantage of transition to living alone may be related to the responsibility for one’s daily life activities and individual independence [23]. Those who live alone need to maintain health-related behaviors and receive additional support through rehabilitation, economic resources, and social networks. Moreover, people with higher socioeconomic status and higher physical and cognitive function would be more likely to live alone; this might explain why older adults living alone had higher MMSE scores than those living with other household members [38,39]. On the other hand, older adults living with other household members had poorer cognitive function and a lower level of independence [11,13] because their household members provided most of the support for them [22].

The study findings also confirm the person–environment fit theory suggesting that older adults with independent living concordance are inclined to have good self-rated health status and life satisfaction [22,24]. Although household interdependence is a prized cultural ideal in Asia, older adults who became alone had a lower chance of cognitive decline. In addition, those who lived alone were free from the unique stress of giving financial or caregiving support to their children [13]. Older adults who live alone need to maintain healthy behaviors, participate in social activities, and take care of themselves on a daily basis in order to maximize the health benefits of living alone. Therefore, living alone might be more suitable for healthy ageing.

With the traditional Chinese belief in filial piety, living with family members remains the preferred living arrangement for most older adults. Most children have the responsibility to take care of their parents and/or grandparents. Although there is a greater chance of stability over time, about one-quarter of Chinese older adults experience one living arrangement transition in a two-year period; this indicates a degree of fluctuation in living arrangements of the oldest-old [40]. Due to rapid socioeconomic development, urbanization, and the one-child policy, the structure of Chinese households has been fundamentally altered. It is necessary for public health practitioners and social workers to educate older adults that living alone might bring health benefits such as better cognitive function. The traditional filial piety should not be a catalyst of cognitive impairment among older adults. Public health practitioners and social workers might also educate other household members to avoid excessive physical support to their elderly parents and/or grandparents for them to maintain their cognitive function and achieve healthy ageing. 

This study has several strengths. First, drawing on longitudinal data, we were able to examine participants who originally lived with other household members and became alone or moved to an institution. Second, using multistate survival analysis, we investigated changes in older adults’ cognitive function. Compared with the traditional Cox regression, the application of multistate survival analysis allowed us to examine the differences in cognitive impairment change over time [34,35]. Studying the impairment change over time is critical for older adults as their cognitive function might change rapidly. Third, the CLHLS secondary dataset has high reliability and validity [31] because all data collectors and investigators received rigorous training prior to collecting information from the participants. The MMSE examination has also been used in other research efforts [8].

This research is not without limitations. First, only a few older adults from our study sample moved to an institution, which might explain the insignificant association observed between moving to an institution and changes of cognitive function in this analysis. We also need to point out more than 90% of older adults continued to live with their household members that they did not change their living arrangement. Therefore, we should be careful about drawing conclusions regarding the discrepancies among the three types of living arrangements. However, according to a study by Lee and colleagues examining the association between living arrangement and sleep among Chinese older adults [27], nearly 81% of the older adults lived with their household members. This could be the result of the traditional belief of filial piety in the Chinese culture that children have the responsibility to take care of their parents/grandparents.

Second, as this study targeted Chinese older adults, its results may not be generalizable to Western populations because traditional Chinese culture heavily emphasizes filial piety and family support. The structural differences across Eastern and Western values may result in discrepant results. However, given that this study targeted Chinese older adults, our findings can be generalized to other places with similar populations like Singapore, Malaysia, or Taiwan.

Third, we were not able to weight measurements employed in our analysis. The sampling weight measurements in the CLHLS dataset are limited to participants’ age, sex, and residence [41], and these measurements do not capture other critical sociodemographic variables. The lack of sufficient weighted measurements might limit the external validity of our findings. However, including weights might increase standard errors in the regression analysis. Fourth, although we used longitudinal data for statistical analysis, the CLHLS questionnaire did not provide the timing and duration of each participant’s living arrangement transition. Discrepancies between longer and shorter duration of new living arrangements could affect older adults’ cognitive function. Further research efforts should attempt to resolve this study limitation by investigating the duration. Last but not least, because we adopted a de-identified and publicly available dataset, CLHLS, we did not include other sensitive underlying factors in our study, such as genetic predisposition. Further research efforts should examine the potential intertwined effects of living arrangement and genetic predisposition on older adults’ cognitive impairment. 

## 5. Conclusions

This research adds to the body of literature investigating living arrangement transition and changes of cognitive function of Chinese older adults over time. We observed that older adults who transitioned to living alone had lower likelihood of deteriorating cognitive function than those who stayed with other household members. We found no statistically significant association with poorer cognitive function for older adults who moved into an institution. These findings might indicate that, as older adults become alone, public health practitioners or social workers may wish to educate them that such living arrangement might be beneficial to their cognitive function. In addition, public health practitioners and social workers need to increase awareness among family members of the need to grant more autonomy to their elderly parents and thereby decrease the prevalence of cognitive impairment among Chinese older adults who live with other household members.

## Figures and Tables

**Table 1:** Sample characteristics of the final study sample: the Chinese Longitudinal Healthy Longevity Survey, 2009–2014 (*n* = 13,851).

	Overall	Normal (25 ≤ MMSE ≤ 30)	Mild (18 ≤ MMSE ≤ 24)	Moderate (10 ≤ MMSE ≤ 17)	Severe (0 ≤ MMSE ≤ 9)
**Primary Predictor**	*n* (%)	*n* (%)	*n* (%)	*n* (%)	*n* (%)
**Living arrangement**					
Stayed with household member(s)	12,876 (92.96)	9747 (75.7)	2270 (17.63)	461 (3.58)	398 (3.09)
Became alone	917 (6.62)	675 (73.61)	166 (18.1)	44 (4.8)	32 (3.49)
Moved to an institution	58 (0.42)	32 (55.17)	16 (27.59)	3 (5.17)	7 (12.07)
**Covariates**					
**Age**					
65–80	7007 (50.59)	6214 (88.68)	694 (9.9)	68 (0.97)	31 (0.44)
81–95	5522 (39.87)	3691 (66.84)	1337 (24.21)	277 (5.02)	217 (3.93)
Above 95	1322 (9.54)	549 (41.53)	421 (31.85)	163 (12.33)	189 (14.3)
**Gender**					
Male	7107 (51.31)	5902 (83.04)	899 (12.65)	168 (2.36)	138 (1.94)
Female	6744 (48.69)	4552 (67.5)	1553 (23.03)	340 (5.04)	299 (4.43)
**Marital status**					
Married	7703 (55.61)	6589 (85.54)	900 (11.68)	126 (1.64)	88 (1.14)
Others	6148 (44.39)	3865 (62.87)	1552 (25.24)	382 (6.21)	349 (5.68)
**Received formal education**					
No	6790 (49.02)	4335 (63.84)	1732 (25.51)	398 (5.86)	325 (4.79)
Yes	7061 (50.98)	6119 (86.66)	720 (10.2)	110 (1.56)	112 (1.59)
**Residential areas**					
Urban	6547 (47.27)	5032 (76.86)	1028 (15.7)	267 (4.08)	220 (3.36)
Rural	7304 (52.73)	5422 (74.23)	1424 (19.5)	241 (3.3)	217 (2.97)
**Smoking status**					
No	10,810 (78.04)	7944 (73.49)	2036 (18.83)	435 (4.02)	395 (3.65)
Yes	3041 (21.96)	2510 (82.54)	416 (13.68)	73 (2.4)	42 (1.38)
**Alcohol use status**					
No	11,125 (80.32)	8217 (73.86)	2068 (18.59)	446 (4.01)	394 (3.54)
Yes	2726 (19.68)	2237 (82.06)	384 (14.09)	62 (2.27)	43 (1.58)
**Number of times suffering from chronic conditions that required inpatient treatments in the past two years**					
None	10,943 (79.01)	8358 (76.38)	1881 (17.19)	380 (3.47)	324 (2.96)
1–2	2530 (18.27)	1830 (72.33)	506 (20)	102 (4.03)	92 (3.64)
Above 2	378 (2.73)	266 (70.37)	65 (17.2)	26 (6.88)	21 (5.56)
**Life satisfaction**					
Good	8727 (63.01)	6800 (77.92)	1473 (16.88)	309 (3.54)	145 (1.66)
Neutral	4350 (31.41)	3279 (75.38)	821 (18.87)	163 (3.75)	87 (2)
Bad	573 (4.14)	369 (64.4)	151 (26.35)	30 (5.24)	23 (4.01)
Not able to answer	201 (1.45)	6 (2.99)	7 (3.48)	6 (2.99)	182 (90.55)
**Health status**					
Good	6762 (48.82)	5437 (80.41)	1033 (15.28)	207 (3.06)	85 (1.26)
Neutral	4909 (35.44)	3708 (75.53)	914 (18.62)	190 (3.87)	97 (1.98)
bad	1977 (14.27)	1302 (65.86)	499 (25.24)	105 (5.31)	71 (3.59)
Not able to answer	203 (1.47)	7 (3.45)	6 (2.96)	6 (2.96)	184 (90.64)
**Sleep quality**					
Good	8889 (64.18)	6882 (77.42)	1475 (16.59)	303 (3.41)	229 (2.58)
Bad	4962 (35.82)	3572 (71.99)	977 (19.69)	205 (4.13)	208 (4.19)
**Wave**					
2009	5053 (36.48)	4038 (79.91)	1015 (20.09)	0 (0)	0 (0)
2012	5053 (36.48)	3746 (74.13)	848 (16.78)	268 (5.3)	191 (3.78)
2014	3745 (27.04)	2670 (71.3)	589 (15.73)	240 (6.41)	246 (6.57)

**Table 2:** Results of the association between living arrangement transition and changes of cognitive function among Chinese older adults, estimated by the multistate Cox regression model: the Chinese Longitudinal Healthy Longevity Survey, 2009–2014.

	Normal/Mild to Moderate/Severe		Moderate/Severe to Moderate/Severe	
**Primary Predictor**	HR	95% CI	HR	95% CI
**Living arrangement**				
Stayed with household member(s)				
Became alone	0.66 **	(0.52, 0.83)	0.91	(0.46, 1.77)
Moved to an institution	0.94	(0.50, 1.77)	2.14	(0.88, 5.20)
**Covariates**				
**Age**				
65–80				
81–95	3.82 **	(3.05, 4.79)	5.31 *	(1.15, 24.47)
Above 95	7.68 **	(5.96, 9.90)	7.00 *	(1.43, 34.33)
**Gender**				
Male				
Female	1.14	(0.97, 1.34)	0.92	(0.51, 1.63)
**Marital status**				
Married				
Others	1.72 **	(1.45, 2.05)	0.79	(0.42, 1.47)
**Received formal education**				
No				
Yes	0.55 **	(0.46, 0.65)	0.50	(0.23, 1.09)
**Residential areas**				
Urban				
Rural	0.99	(0.87, 1.12)	0.89	(0.64, 1.24)
**Smoking status**				
No				
Yes	0.95	(0.78, 1.17)	0.59	(0.28, 1.23)
**Alcohol use status**				
No				
Yes	0.84	(0.69, 1.04)	0.82	(0.38, 1.76)
**Number of times suffering from chronic conditions that required inpatient treatments in the past two years**				
None				
1–2	1.00	(0.85, 1.18)	0.97	(0.64, 1.49)
Above 2	1.33	(0.97, 1.82)	0.93	(0.40, 2.16)
**Life satisfaction**				
Good				
Neutral	1.20 *	(1.01, 1.43)	1.56	(0.85, 2.85)
Bad	1.70 **	(1.22, 2.37)	1.44	(0.50, 4.12)
Not able to answer	3.91 **	(2.09, 7.33)	1.48	(0.28, 7.74)
**Health status**				
Good				
Neutral	1.11	(0.92, 1.32)	0.87	(0.44, 1.75)
Bad	1.63 **	(1.31, 2.03)	1.16	(0.52, 2.58)
Not able to answer	3.25 **	(1.72, 6.16)	2.35	(0.43, 12.76)
**Sleep quality**				
Good				
Bad	1.06	(0.92, 1.22)	1.03	(0.72, 1.46)

* *p*-value < 0.05; ** *p*-value < 0.01.

## Data Availability

The CLHLS dataset is available in the public domain under the National Archive of Computerized Data on Aging (https://www.icpsr.umich.edu/web/NACDA/studies/36692, accessed on 11 September 2021).

## References

[1] HelpAge International Global AgeWatch Insights: The Right to Health for Older People, the Right to Be Counted. http://globalagewatch.org/reports/global-agewatch-insights-2018-report-summary-and-country-profiles/.

[2] Lipnicki D.M., Crawford J., Kochan N.A., Trollor J.N., Draper B., Reppermund S., Maston K., Mather K.A., Brodaty H., Sachdev P.S. (2017). Sydney Memory and Ageing Study Team. Risk Factors for Mild Cognitive Impairment, Dementia and Mortality: The Sydney Memory and Ageing Study. J. Am. Med. Dir. Assoc..

[3] Livingston G., Sommerlad A., Orgeta V., Costafreda S.G., Huntley J., Ames D., Mukadam N. (2017). Dementia prevention, intervention, and care. Lancet.

[4] Petersen R.C., Roberts R.O., Knopman D.S., Boeve B.F., Geda Y.E., Ivnik R.J., Jack C.R. (2009). Mild Cognitive Impairment: Ten Years Later. Arch. Neurol..

[5] The World Health Organization (WHO) Risk Reduction of Cognitive Decline and Dementia: WHO Guidelines. https://www.who.int/mental_health/neurology/dementia/guidelines_risk_reduction/en/.

[6] Kuang W., Gao M., Tian L., Wan Y., Qiu P. (2020). Trends in the prevalence of cognitive impairment in Chinese older adults: Based on the Chinese Longitudinal Healthy Longevity Survey cohorts from 1998 to 2014. Int. Health.

[7] Xu J., Wang J., Wimo A., Fratiglioni L., Qiu C. (2017). The economic burden of dementia in China, 1990–2030: Implications for health policy. Bull. World Health Organ..

[8] An R., Liu G.G. (2016). Cognitive impairment and mortality among the oldest-old Chinese. Int. J. Geriatr. Psychiatry.

[9] Feng Z., Falkingham J., Liu X., Vlachantoni A. (2017). Changes in living arrangements and mortality among older people in China. SSM-Popul. Health.

[10] Evans I., Llewellyn D.J., Matthews F.E., Woods R.T., Brayne C., Clare L. (2019). CFAS-Wales research team. Living alone and cognitive function in later life. Arch. Gerontol. Geriatr..

[11] Imamura H., Uchiyama E., Akiyama M., Kaneko I., Takebayashi T., Nishiwaki Y. (2020). Relationship of living arrangement with the decline in functional capacity in elderly people by gender: A longitudinal observational study. Environ. Health Prev. Med..

[12] Mazzuco S., Meggiolaro S., Ongaro F., Toffolutti V. (2016). Living arrangement and cognitive decline among older people in Europe. Ageing Soc..

[13] Roystonn K., Abdin E., Shahwan S., Zhang Y., Sambasivam R., Vaingankar J.A., Mahendran R., Chua H.C., Chong S.A., Subramaniam M. (2020). Living arrangements and cognitive abilities of community-dwelling older adults in Singapore. Psychogeriatr. Off. J. Jpn. Psychogeriatr. Soc..

[14] Wick J.Y. (2017). Aging in place: Our house Is a very, very, very fine house. Consult. Pharm.®.

[15] Zimmer Z., Yi Z., Poston D.L., Vlosky D.A., Gu D. (2008). Health and Living Arrangement Transitions among China’s Oldest-old. Healthy Longevity in China: Demographic, Socioeconomic, and Psychological Dimensions.

[16] Feng Z., Jones K., Wang W.W. (2015). An exploratory discrete-time multilevel analysis of the effect of social support on the survival of elderly people in China. Soc. Sci. Med..

[17] Wang J., Chen T., Han B. (2014). Does co-residence with adult children associate with better psychological well-being among the oldest old in China?. Aging Ment. Health.

[18] Xu Q., Wang J., Qi J. (2019). Intergenerational coresidence and subjective well-being of older adults in China: The moderating effect of living arrangement preference and intergenerational contacts. Demogr. Res..

[19] Zeng Y., Poston D.L., Vlosky D.A., Gu D. (2008). Healthy Longevity in China: Demographic, Socioeconomic, and Psychological Dimensions.

[20] Zhang L. (2015). Living Arrangements and Subjective Well-Being among the Chinese Elderly. Open J. Soc. Sci..

[21] Ren Q., Treiman D.J. (2015). Living Arrangements of the Elderly in China and Consequences for Their Emotional Well-being. Chin. Sociol. Rev..

[22] Sereny M. (2011). Living Arrangements of Older Adults in China: The Interplay Among Preferences, Realities, and Health. Res. Aging.

[23] Li L.W., Zhang J., Liang J. (2009). Health among the oldest-old in China: Which living arrangements make a difference?. Soc. Sci. Med..

[24] Sereny M.D., Gu D. (2011). Living arrangement concordance and its association with self-rated health among institutionalized and community-residing older adults in China. J. Cross-Cult. Gerontol..

[25] Zhou M., Qian Z. (2008). Social support and self-reported quality of life China’s oldest old. Healthy Longevity in China: Demographic, Socioeconomic, and Psychological Dimensions.

[26] Lund R., Due P., Modvig J., Holstein B.E., Damsgaard M.T., Andersen P.K. (2002). Cohabitation and marital status as predictors of mortality—an eight year follow-up study. Soc. Sci. Med..

[27] Lee Y.-H., Chang Y.-C., Chiang T., Liu C.-T., Shelley M. (2020). Living Arrangements and Sleep-Related Outcomes Among Older Adults in China: A Panel Analytic Approach. Int. J. Aging Hum. Dev..

[28] Ren L., Zheng Y., Wu L., Gu Y., He Y., Jiang B., Zhang J., Zhang L., Li J. (2018). Investigation of the prevalence of Cognitive Impairment and its risk factors within the elderly population in Shanghai, China. Sci. Rep..

[29] Kasper J.D., Pezzin L.E., Rice J.B. (2010). Stability and changes in living arrangements: Relationship to nursing home admission and timing of placement. The Journals of Gerontology. Ser. B Psychol. Sci. Soc. Sci..

[30] Zeng Y. (2012). Towards deeper research and better policy for healthy aging—Using the unique data of Chinese Longitudinal Healthy Longevity Survey. China Econ. J..

[31] Gu D. (2008). General data quality assessment of the CLHLS. Healthy Longevity in China: Demographic, Socioeconomic, and Psychological Dimensions.

[32] Pezzotti P., Scalmana S., Mastromattei A., Di Lallo D. (2008). Progetto Alzheimer Working Group. The accuracy of the MMSE in detecting cognitive impairment when administered by general practitioners: A prospective observational study. BMC Fam. Pract..

[33] Jockusch J., Hopfenmuller W., Nitschke I. (2021). Chewing function and related parameters as a function of the degree of dementia: Is there a link between the brain and the mouth?. J. Oral Rehabil..

[34] Le-Rademacher J.G., Peterson R.A., Therneau T.M., Sanford B.L., Stone R.M., Mandrekar S.J. (2018). Application of multi-state models in cancer clinical trials. Clin. Trials.

[35] Meira-Machado L., de Uña-Alvarez J., Cadarso-Suárez C., Andersen P.K. (2009). Multi-state models for the analysis of time-to-event data. Stat. Methods Med Res..

[36] Therneau T.M. Package “Survival”. https://cran.r-project.org/web/packages/survival/survival.pdf.

[37] Conroy R.M., Golden J., Jeffares I., O’Neill D., McGee H. (2010). Boredom-proneness, loneliness, social engagement and depression and their association with cognitive function in older people: A population study. Psyhology Health Med..

[38] Wang B., He P., Dong B. (2015). Associations between social networks, social contacts, and cognitive function among Chinese nonagenarians/centenarians. Arch. Gerontol. Geriatr..

[39] Yeh S.-C.J., Liu Y.-Y. (2003). Influence of social support on cognitive function in the elderly. BMC Health Serv. Res..

[40] Zimmer Z. (2005). Health and living arrangement transitions among China’s oldest-old. Res. Aging.

[41] Gu D., Sautter J., Pipkin R., Zeng Y. (2010). Sociodemographic and health correlates of sleep quality and duration among very old Chinese. Sleep.

